# A multiplex PCR-based sequencing method for the diagnosis and differentiation of echinococcosis using plasma samples, a proof-of-concept study

**DOI:** 10.1016/j.crmeth.2026.101428

**Published:** 2026-05-07

**Authors:** Yanping Zhao, Yeqin Wang, Ming Zhi, Lin Yang, Wending Pang, Tian Chen, Yiyang Shi, Shu Shen, Hong-Bin Yan, Chunyang Li, Gengfu Wei, Yanyan Zhang, Xin Jin, Yan Zhang

**Affiliations:** 1 BGI Research, Shenzhen 518083, China; 2 BGI Research, Chongqing 401329, China; 3 Department of Hepatobiliary Surgery II, The People’s Hospital of Ganzi Tibetan Autonomous Prefecture, Kangding, Sichuan 626000, China; 4 College of Life Sciences, University of Chinese Academy of Sciences, Beijing 100049, China; 5 School of Biology and Biological Engineering, South China University of Technology, Guangzhou 510006, China; 6 Department of Liver Surgery, West China Hospital of Sichuan University, Chengdu, Sichuan 610041, China; 7 State Key Laboratory for Animal Disease Control and Prevention, Lanzhou Veterinary Research Institute, Chinese Academy of Agricultural Sciences, Lanzhou 730046, China; 8 Biomedical Big Data Center, Med-X Center for Informatics, West China Hospital of Sichuan University, Chengdu, China; 9 State Key Laboratory of Genome and Multi-Omics Technologies, BGI Research, Shenzhen, China; 10 Shenzhen Key Laboratory of Transomics Biotechnologies, Shenzhen, China; 11 Shanxi Medical University-BGI Collaborative Center for Future Medicine, Shanxi Medical University, Taiyuan, China

**Keywords:** *Echinococcus granulosus*, *Echinococcus multilocularis*, echinococcosis, liquid biopsy, target sequencing, non-invasive diagnosis, cell-free DNA

## Abstract

Echinococcosis poses a significant public health threat, and its diagnosis relies on imaging. Non-invasive plasma detection of *Echinococcus* cell-free DNA (cfDNA) offers a promising diagnostic approach. We developed a low-cost multiplex PCR panel with 230 primer pairs targeting *Echinococcus* mitochondrial genomes and nuclear DNA repeat regions. We validated the panel using gradient dilutions of two pathogen species’ DNA and 81 plasma samples (53 patients and 28 controls), differentiating species through sequencing analysis. Our method detected *Echinococcus* genomic DNA at an input as low as 1 fg per reaction, and tests of clinical samples showed a sensitivity of 68.42% and specificity of 92.86%. Based on our detection, 80% of the clinically confirmed cases were correctly identified as positive echinococcosis cases, and cfDNA levels correlated significantly with lesion size. This study highlights the potential of targeted cfDNA sequencing for non-invasive echinococcosis diagnosis and species differentiation and offers a promising tool for doubtful cases.

## Highlights

• A fast and low-cost method for *Echinococcus* cfDNA sequencing using plasma samples
• The lower detection limit of *Echinococcus* DNA is as low as 1 fg/reaction
• *Echinococcus* cfDNA levels significantly correlate with lesion size
• The method enables non-invasive detection of infection and species differentiation

## Motivation

Echinococcosis remains a significant public health threat worldwide. Traditional diagnostic methods for echinococcosis rely heavily on imaging. Previous studies have suggested that sequencing of plasma cell-free DNA (cfDNA) can enhance diagnostic accuracy, but it is both costly and time-consuming. We hypothesized that a low-cost multiplex PCR panel could effectively detect *Echinococcus* cfDNA and differentiate species, improving diagnostic objectiveness, particularly in challenging cases. By exploring the potential of our multiplex panel, we aimed to establish a reliable method for non-invasive echinococcosis diagnosis and species identification.

## Graphical abstract

**Figure:**
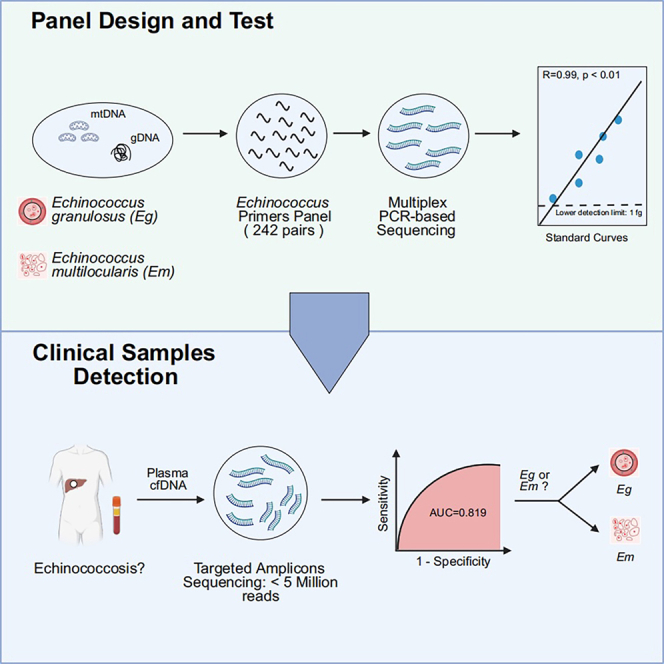

## Teaser

Sequencing of plasma cell-free DNA (cfDNA) can enhance the diagnosis of parasitic infections, but it is usually costly and time-consuming. Zhao et al. develop a fast and low-cost method for *Echinococcus* cfDNA sequencing. The targeted cfDNA sequencing method provides an alternative way to facilitate parasitic infection detection, disease monitoring, and species differentiation.

## Introduction

Globally, alveolar echinococcosis (AE) and cystic echinococcosis (CE) are the 2^nd^ and 3^rd^ most important foodborne parasitic diseases.^1^ As treatment differs between CE and AE, it is essential to make an accurate diagnosis. CE is caused by *Echinococcus granulosus sensu lato*, whose life cycle is more domestic. AE is caused by *Echinococcus multilocularis*, whose life cycle is more sylvatic.^2^^,^^3^ AE is more lethal, as 90% of AE patients die within 10–15 years after diagnosis if left untreated or with inadequate treatment.^4^ Early diagnosis and medical care are important for improving patients’ quality of life.

Before surgery, echinococcosis diagnosis relies on clinical findings, serology, and imaging.^5^ Serology, such as enzyme-linked immunosorbent assays (ELISAs), may yield false positives due to past infection or cross-reaction with other parasites.^6^ Imaging is the basis for clinical diagnosis, the staging of echinococcosis, and the differentiation between AE and CE.^5^ Differentiating AE and CE by imaging is sometimes challenging, especially at an early stage and when the imaging presentation is atypical. Thus, misdiagnosis sometimes occurs.^7^^,^^8^^,^^9^^,^^10^ Besides, approximately 8.6% of echinococcosis patients cannot be classified into CE or AE with the available measures.^9^^,^^11^ Attempts have been made to differentiate occupying lesions caused by CE, AE, and other diseases, often based on surgical biopsy of lesions.^7^^,^^12^^,^^13^ However, echinococcosis patients with small lesions may not need surgical removal, and some severe patients may come to the hospital too late, with main vasculature invasion, and miss the surgical opportunity. In these situations, the pathology reports are unattainable.^4^^,^^14^ An accurate and objective “non-invasive” diagnosis would help doctors take appropriate measures to treat AE or CE patients more effectively.

Plasma cell-free DNA (cfDNA) has been used in prenatal testing and cancer liquid biopsy.^15^^,^^16^ Other groups and we showed that *Echinococcus* cfDNA fragments exist in echinococcosis patients’ blood plasma, through high-depth cfDNA sequencing analysis.^17^^,^^18^^,^^19^^,^^20^^,^^21^ In comparison, qPCR-based methods were less successful in detecting *Echinococcus* cfDNA.^17^^,^^22^ However, the high cost and long turnaround period of untargeted cfDNA sequencing methods hinder its application in clinical settings.

This study aimed to develop a multiplex PCR-based sequencing method to enhance the diagnosis of echinococcosis and differentiate pathogenic species, achieving lower costs and reduced turnaround time.

## Results

### The multiplex PCR panel design and validation process

**Figure 1:**
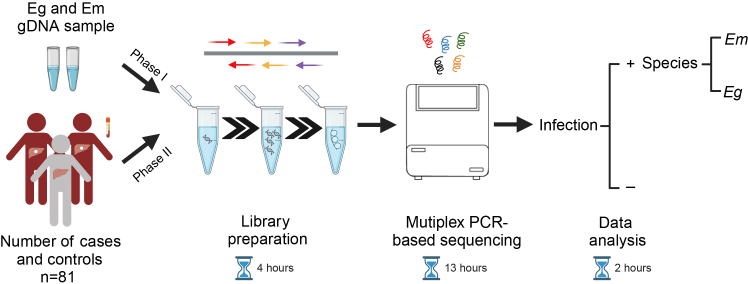
Schematic of the study design In phase I, we used gradient-diluted *E. granulosus* and *E. multilocularis* gDNA to validate the panel performance and identify the detection limit. In phase II, we used 81 plasma samples for real-world validation using clinical samples. *Eg*, *Echinococcus granulosus*; *Em*, *Echinococcus multilocularis*. The figure was drawn with BioRender.

We designed a multiplex PCR panel of 242 targeted amplicons, based on the *E. granulosus* and *E. multilocularis* mitochondrial and nuclear genomes (Table S1) and the primer designs from the literature.^20^^,^^23^^,^^24^^,^^25^ After the panel was ready, we used two phases to validate the panel. The schematic of the study design is shown in Figure 1.

As shown in Figure 1, we first used gradient-diluted *E. granulosus* and *E. multilocularis* genomic DNA (gDNA) to validate the panel performance and identify the detection limit. After that, we used 81 plasma samples from our prospective cohort, including patients and controls, for real-world validation in a phase II clinical setting. The whole detection process took approximately 19 h, including library preparation, multiplex PCR-based sequencing, and data analysis. The data analysis involved two steps, with step 1 to determine whether the sample was *Echinococcus*-infected (positive) according to either the comparison to the negative control (phase I) or clinical controls (phase II). In step 2, we used the proportion of *Echinococcus* reads in the detected positive sample to determine whether this sample was infected with *E. multilocularis* or *E. granulosus* (AE and CE, respectively).

### The multiplex PCR-based sequencing method detected *Echinococcus* gDNA input as low as 1 fg

**Figure 2:**
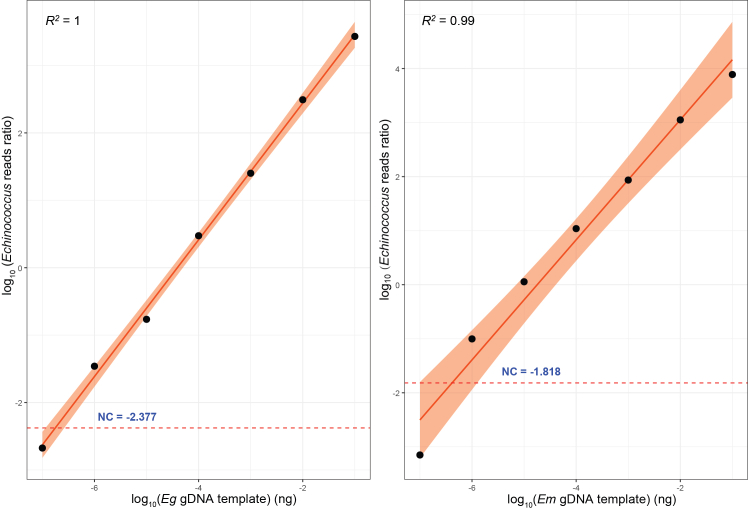
Validation and detection limit determination using diluted *Echinococcus* gDNA samples Two technical replicates for *E. granulosus* and *E. multilocularis* gDNA libraries were sequenced. The red line is the regression line based on a regression model (with 95% confidence intervals shown as shaded areas). The R is the Pearson correlation coefficient. (A) *E. granulosus* serial dilution curve. The *x* axis indicates the mean amount of *E. granulosus* gDNA template input (ng) (log_10_) in the two replicates, and the *y* axis is the mean of the *Echinococcus* read ratio (to the Lambda DNA reads) in the two replicates (log_10_). (B) *E. multilocularis* serial dilution curve. The *x* axis indicates the mean of the *E. multilocularis* gDNA template input (ng) (log_10_) in the two replicates, and the *y* axis is the mean of the *Echinococcus* read ratio (to the Lambda DNA reads) in the two replicates. *Eg*, *Echinococcus granulosus*; *Em*, *Echinococcus multilocularis*; NC, negative control. See also Figure S1.

The standard curves of this panel using *E. granulosus* and *E. multilocularis* gDNA as PCR templates are shown in Figure 2. The detailed genomic positions and the primer performance of the 242 targeted amplicons (including 12 amplicons for quality control) can be found in Tables S1 and S2, respectively. The 210 primer pairs targeting mitochondrial DNA regions exhibited high performance, achieving 100% target coverage with all sequencing depths >100X (see STAR Methods; Figure S1). The primer pair targeting a specific nuclear repeat region designed in this study also showed excellent performance. Six of the literature primer pairs worked, but 13 pairs did not (highlighted in bold in Table S2).

The serial dilution curves of *Echinococcus* gDNA, ranging from 0.1 ng to 0.1 fg, demonstrated a strong positive correlation between the input of *Echinococcus* gDNA and the *Echinococcus* reads ratio (defined as the reads ratio of *Echinococcus* to the spike-in of Lambda DNA). At the input level of 0.1 fg, the number of *Echinococcus* reads ratio was lower than that of the negative control samples (dotted lines in Figure 2); thus, the lower detection limit was about 1 fg for both the *E. multilocularis* and *E. granulosus* gDNA samples, corresponding to approximately 0.005 genome equivalents (GE) of *E. multilocularis* and 0.008 GE of *E. granulosus*, respectively, which is much lower than detection limit of conventional quantitative PCR methods.

### Clinical patients and plasma samples

We used 81 plasma samples (53 patients and 28 clinical controls, 0.2 mL/sample) along with the positive control of *Echinococcus* gDNA to validate our panel for identifying *Echinococcus* infection and differentiating between AE and CE. The participants’ demographics are shown in Table 1 (individual patient details in Table S3). The cases and controls were not significantly different in age (*p* = 0.43) (see STAR Methods; Figure S2) or sex ratio (*p* = 0.09).

**Table 1:** Summary of the sample used (including participants’ demographics)

	Number	%
**Sample type**		

Plasma	81	91.01
*Echinococcus* gDNA	8	8.99

**Sex**		

Male	33	40.74
Female	48	59.26

**Age (years)**		

Maximum	67	NA
Minimum	8	NA
Mean	38 (SD 14)	NA

**Case/control**		

Case	53	65.43
Control	28	34.57

**AE/CE in cases**		

AE	31	58.49
CE	22	41.51

**Before surgery**		

Before AE surgery	31	100
Before CE surgery	7	31.82

**Panel positive among samples before surgery**		

+ Before AE surgery	21	67.74
+ Before CE surgery	5	71.43

**Positive among other samples**		

+ Non-echinococcosis plasma samples	2	7.14
+ *Echinococcus* gDNA samples	8	100.00

**Lesion size (mm)**		

Maximum	196	NA
Minimum	10	NA
Mean	80.81 (SD 38.91)	NA

**Lesion size (mm) in AE patients**		

Maximum	196	NA
Minimum	30	NA
Mean	85.10 (SD 44.80)	NA

**Lesion size (mm) in CE patients**		

Maximum	145	NA
Minimum	10	NA
Mean	75.09 (SD 28.49)	NA

**WHO classification in AE**		

P1N0M0	17	54.84
P2N0M0	7	22.58
P3N0M0	3	9.68
P2N1M0	2	6.45
P3N1M0	2	6.45

**WHO classification in CE**		

Missing	1	4.55
CE2	12	54.54
CE3	3	13.64
CE4	4	18.18
CE5	1	4.55
CE2, CL	1	4.55

NA, not applicable; SD, standard deviation; AE, alveolar echinococcosis; CE, cystic echinococcosis.

The detailed information of the sample, including the individual participants, is shown in Table S3.

All 53 echinococcosis patients tested positive by ELISA. Among them, 50 patients were confirmed by pathological diagnosis, and the other three non-surgical patients were diagnosed by imaging (Table S3). The plasma samples of 38 patients (31 AE and 7 CE patients) were collected before surgery. The above-mentioned 28 clinical controls included 24 relatives of echinococcosis patients without any clinical symptoms of *Echinococcus* infection and four non-echinococcosis patients (Table S3).

### *Echinococcus* cfDNA detection and the differentiation between AE and CE patients before surgery

**Figure 3:**
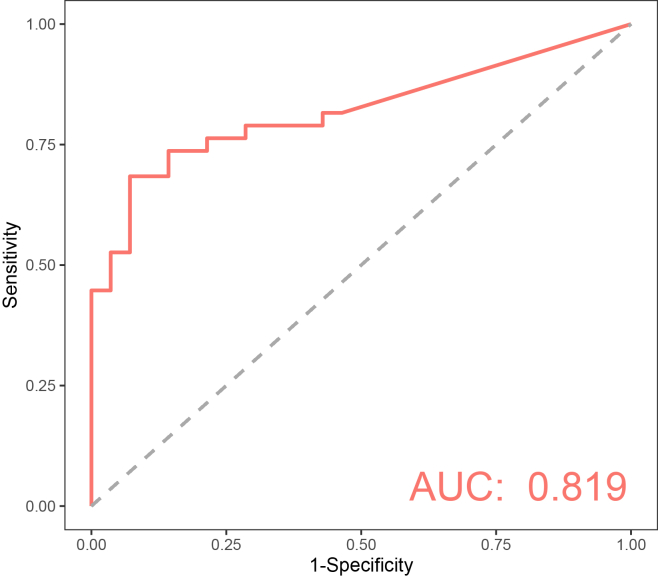
ROC curves for all echinococcosis patients before surgery (38) and the clinical controls (28) AUC, area under the curve. See also Figure S3.

Based on the *Echinococcus* fraction (%) determined by our analysis methods, the receiver operating characteristic (ROC) curves were drawn using 38 patient samples (31 AE and 7 CE) before surgery and the 28 clinical controls (Figure 3). Postsurgical plasma samples were not included in this analysis because theoretically, the surgical excision of the lesion should lead to the clearance of *Echinococcus* cfDNA. A heatmap showing the sequencing depth of target amplicon regions is also presented (see STAR Methods; Figure S3). The sample with the highest *Echinococcus* fraction was GZ-463 (lesion size = 165 mm), from an AE patient at an advanced stage who lost surgical opportunities. Multiple amplicon regions showed consistently high sequencing depths for samples with relatively high *Echinococcus* fraction (see STAR Methods; Figure S3).

As shown in Figure 3, the area under the curve (AUC) was 0.819 for all echinococcosis patients’ samples before surgery, with a 95% confidence interval (CI) of 0.719–0.919. At an *Echinococcus* fraction (%) cut-off value of 0.1178%, the ROC curve for all echinococcosis reached a Youden index of 0.613. At this cut-off value, only two of the 28 control samples were identified as positive, and the calculated specificity was 92.86%. All four samples from pathologically confirmed non-echinococcosis patients in the clinical control group (Table S3) were detected as negative by our method. Meanwhile, the overall sensitivity was 68.42%. Notably, the sensitivity was much higher for the patients with larger lesions, which reached 92.86% and 88.24% for patients with lesions over 84.94 mm (mean) and 70.00 mm (median), respectively.

**Figure 4:**
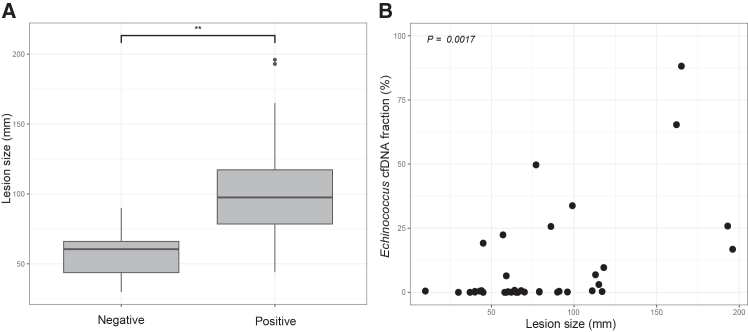
Relationship between lesion size and test results among the samples before surgery (A) Lesions in the positive group were significantly larger than those in the negative group. Box plots show median ± interquartile range (IQR) and 1.5 IQR ranges (whiskers). (B) Correlation between the *Echinococcus* cfDNA fraction and lesion size. Spearman’s rank correlation coefficient (Spearman’s rho = 0.493).

To clarify the influence of the lesion sizes, we also compared the identified positive and negative patients (before surgery) according to our method. As shown in Figure 4A, the lesion sizes of the positive group (*n* = 26, median = 82.50 mm) were significantly larger than those of the negative group (*n* = 12, median = 60.50 mm) (*p* < 0.05, Mann-Whitney *U* test). Then, we tested the correlation between lesion size and the *Echinococcus* cfDNA fraction (Figure 4B). The normalized *Echinococcus* cfDNA fraction was significantly correlated with lesion size (*p* < 0.01, Spearman’s rho = 0.493; Figure 4B).

Using 0.1178% as the cut-off value, 21 of the 31 AE patient samples were identified as positive, among which 17 (80.95%) were accurately identified as infected by *E. multilocularis* (AE) and four (19.05%) were identified as infected by *E. granulosus* (CE). Besides, five of the seven CE samples before surgery were identified as positive, of which four were correctly identified as CE. For the two CE samples that could not be identified by our method, one was from a CE2 patient (GZ-098) with approximately 4 years of albendazole treatment, and the other was from a CE4 patient (GZ-097), an inactive stage of the cyst.^5^

### *Echinococcus* cfDNA was not detected in the plasma of most patients after surgery

In this study, we included 14 CE patients who had undergone surgery for lesion removal, with their blood samples collected 1–9 days post-surgery. Among the 14 samples, 11 were identified as negative (including all samples from stage CE4 and CE3 patients), while the other three samples were identified as positive (Table S3) and correctly identified as *E. granulosus* rather than *E. multilocularis* infection through our sequence analysis. These positive results may indicate residual lesions in the 3 patients after surgery. The fact is true for a special CE patient (GZ-236), who had undergone a liver lesion removal operation 6 years before and then relapsed with a lesion size of 50 mm (Table S3). All CE plasma samples collected after surgery were positive according to ELISA. The long-term existence of antibodies in the human body makes the ELISA-based serological assay less informative for disease monitoring in patients after surgery. In comparison, our method overcomes the problem through real-time monitoring of the *Echinococcus* cfDNA in the patients, with the half-life of plasma cfDNA rather short, only about an hour or less.^26^

**Figure 5:**
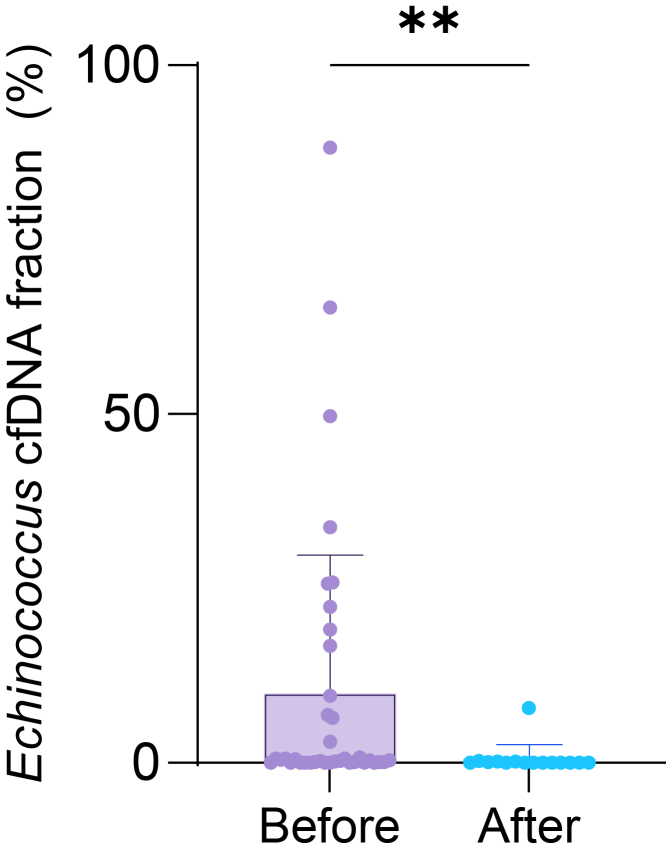
*Echinococcus* cfDNA fraction comparison between echinococcosis patient samples before (38, including 31 AE and 7 CE) and after surgery (15 CE and 0 AE). Box plots show median ± interquartile range (IQR) and 1.5 IQR ranges (whiskers).

We compared the *Echinococcus* cfDNA fractions of echinococcosis patient samples before and after surgery (Figure 5). As shown in Figure 5, the *Echinococcus* cfDNA fraction in samples before surgery (median = 0.445%) was significantly greater than that after surgery (median = 0.022%) (*p* < 0.005, Mann-Whitney *U* test).

In this study, two patients (GZ-257 and GZ-189) had previously been misdiagnosed as AE patients according to the imaging results before surgery, but later on, pathologic results confirmed GZ-257 as a liver cancer patient and GZ-189 as a CE patient. In our detection method, we correctly identified GZ-257 as negative. For GZ-189, we only obtained post-surgical blood, and this sample was found to be negative by our method.

## Discussion

We developed and validated a multiplex PCR-based sequencing method to identify plasma *Echinococcus* cfDNA to help diagnose and differentiate echinococcosis. More objective diagnosis before surgery could improve the management of echinococcosis patients, especially in epidemic regions with multiple *Echinococcus* species. As far as we know, this is the largest multiplex PCR panel for *Echinococcus* cfDNA detection and a comprehensive cfDNA panel to differentiate between AE and CE patients. The detection limit was as low as 1 fg of *Echinococcus* gDNA per reaction, which corresponds to less than 0.01 GE, and the detection limit is at least two orders of magnitude lower than the conventional quantitative PCR methods targeting a regular nuclear DNA region (>1 copy/reaction).

The sensitivity of our panel was 68.42%, and the specificity was 92.86% using clinical plasma samples. This performance was somewhat similar to early cancer detection using cfDNA.^27^^,^^28^ Based on our standard curve and the lower detection limit analysis, we believe that the very tiny amount of *Echinococcus* cfDNA released into the blood is the major limiting factor for detection. In comparison, the conventional qPCR methods with only one or two target amplicons often result in a sensitivity of less than 23.8% for plasma samples.^29^^,^^30^ With 16 PCR primer pairs, a sensitivity of 62.5% (15/24) was reported, using samples of advanced echinococcosis patients.^20^ Unfortunately, no lesion size information was provided in that study, which made the direct comparison with our results difficult, and our findings and previous literature indicated that the cfDNA in plasma was positively related to lesion size.^18^

In previous work, using the untargeted cfDNA sequencing method with a very large amount of sequencing data (∼253 million clean reads), the sensitivity reached 62.5%–100%.^17^^,^^18^^,^^19^^,^^20^ However, these studies often used 10 mL blood,^18^^,^^20^^,^^31^ with the sample volumes much larger than that of 0.4 mL of blood (∼0.2 mL plasma) in our study. Untargeted high-depth cfDNA sequencing is too costly and time-consuming for clinical use. Our method reduced both the cost and time for sequencing and analyzing. The clean reads were only about 2.6 million per sample, approximately 1% of the reads needed for untargeted cfDNA sequencing (253 million).^19^ As a result, the bioinformatic analysis is much faster with even lower computational equipment requirements, making it much easier to fit in a clinical setting. Moreover, the results can be obtained within 20 h if a flexible and fast sequencing platform were used for targeted sequencing, such as G99 (MGI, Shenzhen, China).

Previous cfDNA-based methods cannot differentiate between AE and CE. Some published research mainly studied plasma samples from CE patients,^19^^,^^21^ or mainly studied plasma samples from AE patients and set CE patients as controls.^18^^,^^31^ Here, we demonstrated the potential of using *Echinococcus* cfDNA to differentiate between AE and CE patients before surgery. This non-invasive liquid biopsy test could provide objective clues for managing all echinococcosis patients, especially complicated patients. Traditionally, more objective differentiation relies on biopsy materials obtained during surgery, which cannot provide guidance ahead of surgery and cannot be applied to patients whose lesions are inoperable due to their small size or to patients with main vasculature invasion, thus too late for surgery.^7^^,^^12^^,^^13^^,^^14^

Most of the targets in our panel were designed for the mitochondrial DNA, as it has high copy numbers in cells. We pointed out that the primary design was based on mitochondrial DNA without the need for chromosome sequences, which will simplify the design process and may be more feasible for some specific parasites without full knowledge of genomes. Some of the primers targeting nuclear repeated genomic regions from the literature worked well in our panel, while others did not work even with *Echinococcus* gDNA (Table S2), which could be related to the primer design and the compatibility with our panel. Moreover, our input requirement was only 0.2 mL of plasma per sample, which is more feasible than the 10 mL of blood samples used before.^18^^,^^20^^,^^31^ Or alternatively, more target amplicons and a larger blood volume for the test could further increase the detection sensitivity of this method.

### Limitations of the study

Admittedly, one major limitation is that our sample size is not very large, which is a common problem for many echinococcosis diagnostic studies, involving even smaller sample sizes with fewer than 30 patients.^17^^,^^19^^,^^20^^,^^21^^,^^25^^,^^29^^,^^31^^,^^32^ Few studies have enrolled 50 to 105 echinococcosis patients.^18^^,^^33^^,^^34^ We enrolled 53 echinococcosis patients, a comparatively large sample size in echinococcosis diagnostic studies, while the number of CE patients before surgery was relatively small, which is mainly due to the lower prevalence of CE than AE in our sampling area of the Tibetan Plateau in Sichuan Province.^11^^,^^35^

Second, we did not use the ideal clinical control samples. Previous studies used plasma samples from non-epidemic areas as controls.^19^^,^^20^ We used echinococcosis patients’ relatives and patients with other diseases in the same region, which is a more real-world situation in echinococcosis treatment centers. Although plasma samples from non-epidemic patients are better negative controls, the real-world application of the diagnostic tools would be in epidemic regions. Some of these “healthy” relatives might have early infections with no obvious lesions on imaging, as they lived together with the echinococcosis patients and ate the same food. We could not exclude the possibility of early infections beyond the detection limit of the current clinical diagnostic method. Lacking an ideal reference standard is a common problem faced by cfDNA early screening studies, which requires a long-term follow-up to draw conclusions.^36^ We recommend that those relatives with positive results according to our method receive annual ultrasound screenings in the following years.

A similar suggestion can be given to those patients whose postsurgical plasma samples are still positive for *Echinococcus* cfDNA. Previously, high relapse rates were reported among CE patients after surgery.^33^ In our study, we had two patients, GZ-214 and GZ-236, whose lesions were removed 10 and 6 years ago, and both relapsed. In a previous study of AE, patients with *Echinococcus* cfDNA identified after surgery were reported to experience relapse.^18^ CfDNA is a promising indicator of relapse, much better than ELISA, as ELISA results were all positive for the plasma samples after surgery. Previous studies also showed that ELISA provides false positive results after surgery.^6^ Annual ultrasound screening for postsurgical patients with positive *Echinococcus* cfDNA may help to identify possible reinfections/relapses earlier and avoid serious consequences. This finding should be validated in the follow-up study and in a larger cohort study.

In summary, we developed and validated a fast and low-cost multiplex PCR-based sequencing method and strategy that can help facilitate echinococcosis diagnosis, disease monitoring, and differentiation between AE and CE. This proof-of-concept study provides an alternative way for the non-invasive detection of parasitic infections.

## Resource availability

### Lead contact

Requests for further information and resources should be directed to and will be fulfilled by the lead contact, Yan Zhang (zhangyan15@genomics.cn).

### Materials availability

This study did not generate new, unique reagents.

### Data and code availability

• The data reported in this study are stored in the CNGB Nucleotide Sequence Archive (CNSA: https://db.cngb.org/cnsa with accession number CNP0005472).
• This paper does not report original code.
• Any additional information required to reanalyze the data reported in this work paper is available from the lead contact upon request.

## Acknowledgments

This work was supported by the 10.13039/501100004829Science & Technology Department of Sichuan Province funding project (nos. 2020YFS0576 and 2022YFS0091), the 10.13039/501100016107Science and Technology Program of Tibet Autonomous Region (XZ202303ZY0008G), and the 10.13039/501100017610Shenzhen Science and Technology Program (SYSPG20241211173852024).

## Author contributions

Y. Zhao, Y.W., M.Z., L.Y., W.P., T.C., C.L., G.W., Y.Y.Z., Y. Zhang, and X.J., conceived and designed the experiments, carried out the study, including questionnaire designing, data, and sample collection; Y.W., M.Z., and L.Y., performed the experiments; Y. Zhao, Y.W., W.P., L.Y., T.C., Y.S., S.S., H.Y., and Y. Zhang, analyzed the data; and Y. Zhao, Y.W., M.Z., L.Y., W.P., T.C., C.L., G.W., Y.Y.Z., Y. Zhang, and X.J. drafted the manuscript.

## Declaration of interests

Y.W., Y. Zhao, Y. Zhang, H.Y., W.P., and X.J. have filed patent applications for this study.

## STAR★Methods

### Key resources table

**Table:** 

REAGENT or RESOURCE	SOURCE	IDENTIFIER
**Biological samples**		

Human DNA (NA12878)	CORIELL INSTITUTE	Cat#NA12878
Lambda DNA	NEB	Cat#N3011S
*Echinococcus* gDNA	Lanzhou Veterinary Research Institute, Chinese Academy of Agricultural Sciences, Lanzhou, China	N/A
Human plasma samples	The People’s Hospital of Ganzi Tibetan Autonomous Prefecture, Kangding, Sichuan Province, China	N/A

**Critical commercial assays**		

Diagnostic Kit for IgG Antibody to Hydatid (ELISA)	Haitai Biotech	Cat#20153400132
MagPure Circulating DNA KF Kit	Magen	Cat#MD5432-02
ATOPlex DNA Dual BC Library Prep Set	MGI	Cat#940-001191-00
Qubit dsDNA HS Assay Kit	Invitrogen	Cat#Q32854

**Deposited data**		

Sequencing data	This paper	CNSA: https://db.cngb.org/cnsa with accession number CNP0005472

**Oligonucleotides**		

Primer pairs and targeting genomic positions of the amplicons, see Tables S1 and S2.	This study and primers from the literature (Wan, Z. et al.^20^ Spotin, A. et al.,^23^ Kinkar, L. et al.,^24^ Toribio, L. et al.^25^	N/A

**Software and algorithms**		

ATOPlex Designer	MGI	https://atoplex.mgi-tech.com/
SOPAnuke v2.1.0	Chen, Y. et al.^37^	https://github.com/BGI-flexlab/SOAPnuke
Fastp v0.23.2	Chen, S. et al.^38^	https://github.com/OpenGene/fastp
Kraken v1.0	Wood, D.E. et al.^39^	https://ccb.jhu.edu/software/kraken/
Snap-aligner v1.0beta.23	N/A	https://github.com/amplab/snap
Prinseq v0.20.4	N/A	(https://github.com/uwb-linux/prinseq
BWA v0.7.19	Li, H. et al.^40^	https://github.com/lh3/bwa
Samtools	Danecek, P. et al.^41^	http://samtools.sourceforge.net/
Bedtools v2.31.0	Quinlan, A.R. et al.^42^	https://github.com/arq5x/bedtools2
R v4.3.0	R Project	https://www.r-project.org/
Stata	Stata Corp, College Station, TX, USA	https://www.stata.com/
Biorender.com	Biorender	https://www.biorender.com/

### Experimental model and study participant details

#### Ethics approval and consent to participate

The study was approved by the Ethical Committee of West China Hospital of Sichuan University 2020 (773), the People’s Hospital of Ganzi Tibetan Autonomous Prefecture (GZZYY-2020-16), and BGI Research (BGI-IRB 20135-T3). Informed consents from the participants or their guardians were obtained at the time when they agreed to participate. The participants’ demographic information can be found in Table S3.

#### Biological samples and clinical information collection

Lanzhou Veterinary Research Institute provided *Echinococcus* gDNA. Plasma was obtained from a prospective cohort from the People’s Hospital of Ganzi Tibetan Autonomous Prefecture (Chinese Clinical Trial Registry registration: ChiCTR2200060516). The plasma samples from echinococcosis patients and their relatives were collected at Echinococcosis Treatment Center between 2021 and 2023. Pregnant or breastfeeding women were excluded. All the participants were screened with ultrasound, and their plasma was tested with ELISA (Haitai Biotech). The final diagnosis was based on the biopsy pathology report for the patients who underwent surgery, or based on imaging for non-surgical patients. The clinical control included relatives of echinococcosis patients without any clinical symptoms of *Echinococcus* infection and pathologically-confirmed non-echinococcosis patients (Table S3).

### Method details

#### The panel design

We developed a multiplex panel using the ATOPlex Designer (https://atoplex.mgi-tech.com/), incorporating 230 amplicons for *E. granulosus* and *E. multilocularis*. We designed 70–90 bp amplicons including: a) 110 and 100 amplicons for the *E. granulosus* and *E. multilocularis* mitochondria genomes, respectively, given the high copies of the mitochondrion,^23^ b) one amplicon for a repeat genomic region of *E. multilocularis* based on the analysis of untargeted cfDNA sequencing data, c) 19 amplicons of U1 snRNA, *nad5*, and other repeated genomic regions according to prior studies (12 amplicons for *E. granulosus* and 7 amplicons for *E. multilocularis*).^20^^,^^25^^,^^29^^,^^32^ See Tables S1 and S2. The panel used Lambda DNA as a spike-in control (NEB, N3011S) with 1,000 copies/reaction (5 primer pairs), and human genomic DNA from cell line (GM12878) as an internal quality control (1 ng/reaction) (7 primer pairs).

#### Amplicon library preparation and sequencing

*E. granulosus* and *E. multilocularis* gDNA were gradient-diluted to a range from 0.1 ng to 0.1 fg using nuclease-free water (Invitrogen, cat #AM9930) in a 10-fold gradient. Plasma sample of 0.2 mL was used for cfDNA extraction using the MGISP-960 automated preparation platform (MGI, Shenzhen, China) and a magnetic bead-based circulating DNA extraction kit (MagPure Circulating DNA KF Kit, MD5432-02, Magen). Amplicon sequencing library preparation used ATOPlex DNA Dual BC Library Prep Set (940-001191-00, MGI, Shenzhen, China). The experiments followed the instructions and the two-step amplification protocol was set as 35 (13 + 22) PCR cycles. The concentrations of gDNA, extracted cfDNA, and amplified products were measured using Qubit dsDNA HS Assay Kit (Q32854, Invitrogen) and the Qubit 3.0 Fluorometer (Invitrogen). All libraries were sequenced on the DNBSEQ platform using a paired-end 100 bp strategy, with an average of 4.4 million raw reads/sample. Technical replicates of the gDNA libraries were prepared and sequenced.

#### Sequencing reads analysis workflow

The analysis was modified from our previous WGS cfDNA workflow.^19^ Raw data were processed using SOAPnuke v2.1.0 and Fastp v0.23.2. Clean data were processed using Kraken v1.0 with the “-paired” option against the “*Echinococcus*” database established before.^19^ Human reads were removed using Snap-aligner v1.0 beta.23 with a “paired” option against the human reference GRCh37/hg19. Low-complexity reads were filtered using Prinseq v0.20.4 (https://github.com/uwb-linux/prinseq). Clean *Echinococcus* reads were processed against the primer database using BWA-MEM to remove primer dimers.

We calculated the *Echinococcus* to lambda reads ratio from the same reaction to evaluate panel performance. The multiplex PCR panel’s detection limit was defined as the *Echinococcus* gDNA level in the serial dilutions at which the *Echinococcus* to lambda reads ratio was equal to or lower than that in the negative control of TE (Invitrogen, AM9858). We summarised each primer’s performance using 0.1 ng of *Echinococcus* gDNA as input, see Table S2.

We normalised the fractions of *Echinococcus* cfDNA reads to the total number of *Echinococcus* and lambda DNA reads for comparison to avoid the influence of sequencing depth on samples. The normalised *E. multilocularis* and *E. granulosus* fractions were used to differentiate between AE and CE.

$$Echinococcus\;fraction\;\left(\%\right)=\frac{Echinococcus\;reads}{Echinococcus\;reads+Lambda\;DNA\;reads}\times 100$$

$$E.multilocularis\;fraction\;\left(\%\right)=\frac{E.\;multilocularis\;reads}{Total\;Echinococcus\;reads}\times 100$$

$$E.\;granulosus\;fraction\;\left(\%\right)=\frac{E.\;granulosus\;reads}{Total\;Echinococcus\;reads}\times 100$$

The number of genome equivalents (GE) in a reaction was calculated according to the genome size, gDNA concentration, and the input volume.

### Quantification and statistical analysis

The demographics, clinical information, and cfDNA results were analyzed using Stata 14.1 (Stata Corp, College Station, TX, USA). We used the two-sample Mann-Whitney U test to compare the values of two groups of numerical data and the chi-square test for groups of categorical data. Spearman’s correlation coefficients were used to measure the relationship between *Echinococcus* cfDNA fractions and lesion sizes. The primer amplicon unique region depth information was obtained by bedtools v2.31.0 with the “coverage-mean” flag for counting. Receiver operating characteristic (ROC) curves were generated using pROC v1.18.5 based on R version 4.3.0. The study workflow was created using BioRender.com. The other figures were drawn using ggplot2 v3.4.3 or Stata 14.1 (Stata Corp, College Station, TX, USA).

## Supplemental information

Document S1. Figures S1–S3

Table S1. Primer pairs and targeting genomic positions of the amplicons, related to STAR Methods

Table S2. Primer performance for *Echinococcus* gDNA (0.1 ng) replicates, related to STAR Methods

Table S3. Sample and individual details, related to Table 1 and STAR Methods

Document S2. Article plus supplemental information
