# Supplementary material for: A multiplex PCR-based sequencing method for the diagnosis and differentiation of echinococcosis using plasma samples, a proof-of-concept study
Source: Cell Rep Methods. 2026 May 7;6(6):101428. doi: 10.1016/j.crmeth.2026.101428 (PMC13282652; doi:10.1016/j.crmeth.2026.101428)
Supplement: Document S1. Figures S1–S3 [file mmc1.pdf]

Cell Reports Methods, Volume *6*

Supplemental information

A multiplex PCR-based sequencing method for the diagnosis and differentiation of echinococcosis using plasma samples, a proof-of-concept study

Yanping Zhao, Yeqin Wang, Ming Zhi, Lin Yang, Wending Pang, Tian Chen, Yiyang Shi, Shu Shen, Hong-Bin Yan, Chunyang Li, Gengfu Wei, Yanyan Zhang, Xin Jin, and Yan Zhang

SUPPLEMENTAL INFORMATION

Figures S1–S3

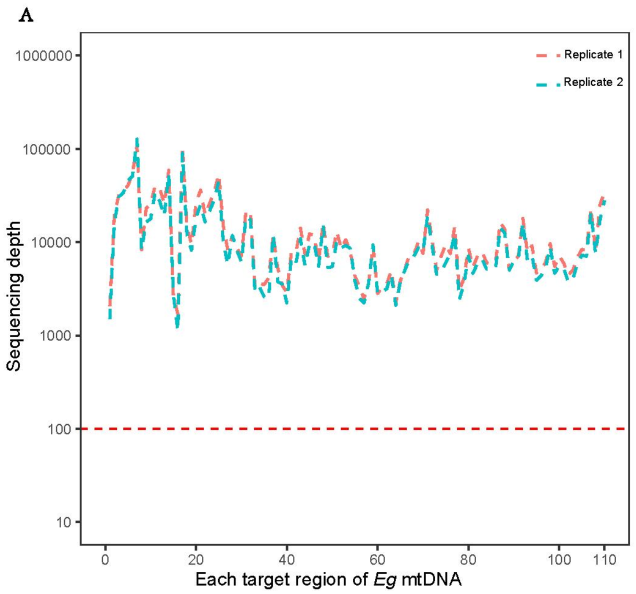

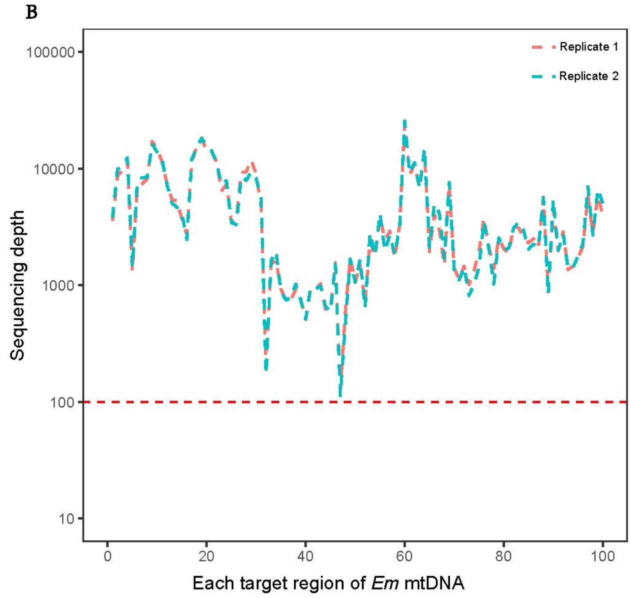

Figure S1. Amplicon sequencing depth in *Echinococcus* gDNA (0.1 ng) replicates, related to Figure 2. (A), *Eg*: *Echinococcus granulosus*. (B), *Em*: *Echinococcus multilocularis*.

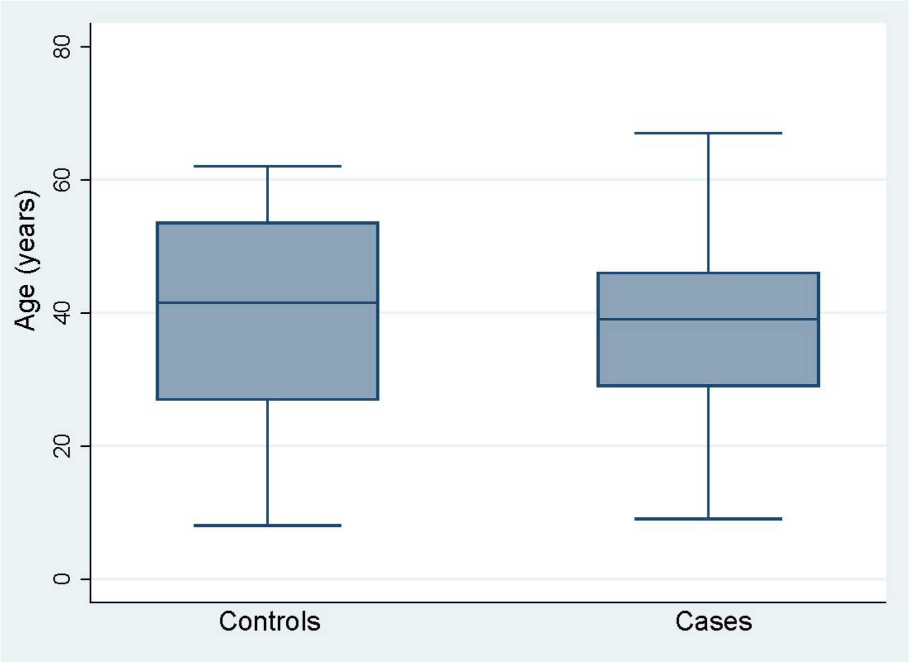

Figure S2. No significant difference in age was found between the patients and controls (P = 0.43), related to Table 1.

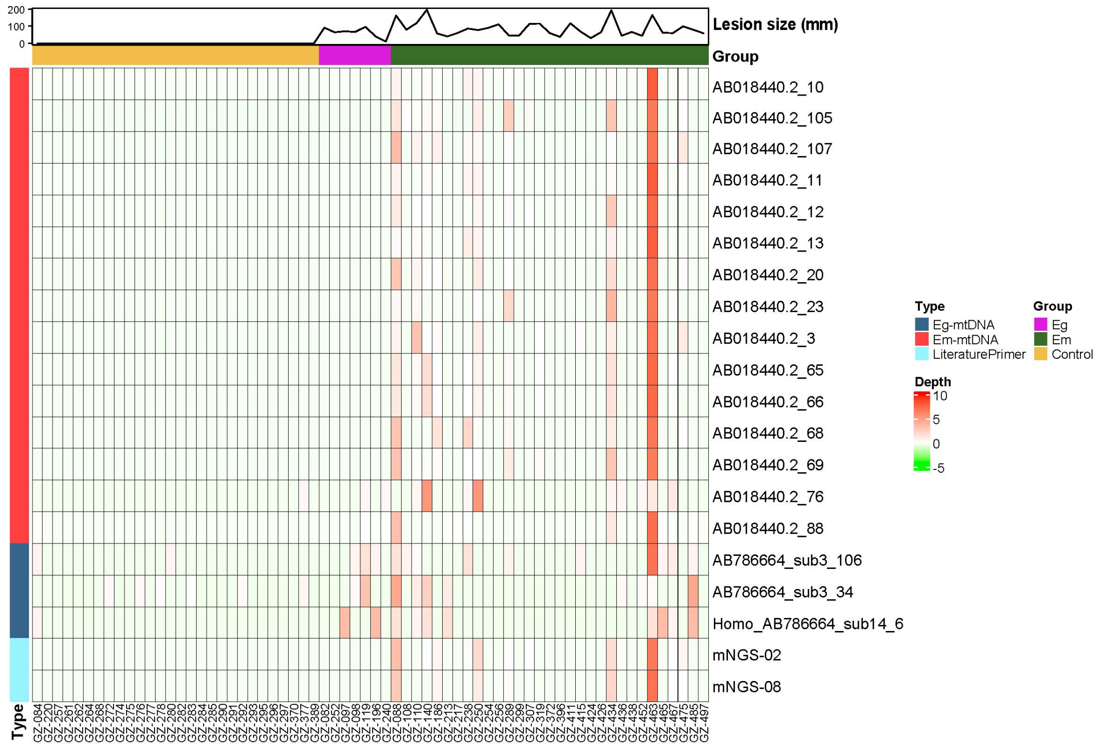

Figure S3. Heatmap visualisation of the sequencing depth of a subset of the amplicons for 38 echinococcosis patients (31 AE and 7 AE) before surgery and 28 clinical controls, related to Figure 3 . The upper annotation shows the maximum diameters of lesions from the patients. The x-axis represents the sample ID. The left colour of the y-axis represents the types of primers. On the right are the primer names, and "Homo" indicates that the primer is a homologous sequence region between *E. multilocularis* and *E. granulosus*. The R package pheatmap was used and scaled in the row direction.
